# Adjusting CA19-9 values with clinical stage and bilirubin to better predict survival of resectable pancreatic cancer patients: 5-year-follow-up of a single center

**DOI:** 10.3389/fonc.2022.966256

**Published:** 2022-07-29

**Authors:** Zuowei Wu, Pengcheng Zhao, Zihe Wang, Xing Huang, Chao Wu, Mao Li, Li Wang, Bole Tian

**Affiliations:** Department of Pancreatic Surgery, West China Hospital, Sichuan University, Chengdu, China

**Keywords:** carcinoma, pancreatic ductal, pancreatectomy, prognosis, biomarkers, CA19-9 antigen, bilirubin

## Abstract

**Background:**

Pancreatic cancer mortality is growing every year, and radical resection is the most essential therapy strategy. It is critical to evaluate the long-term prognosis of individuals receiving radical surgery. CA19-9 is a biomarker for patient recurrence and survival, however obstructive jaundice has a significant impact on this index. Researchers have attempted to modify the index using various modification methods, but the results have been unsatisfactory. In this study, we adjusted CA19-9 values based on clinical stage and bilirubin and found that it provided better prediction than CA19-9 alone in assessing patients.

**Methods:**

We analyzed over 5 years follow-up records of patients who underwent radical pancreatic cancer surgery between August 2009 and May 2017 in a single center. We investigated the association of risk factors with overall survival (OS) as well as disease-free survival (DFS) after surgery. Threshold values for high-risk features associated with poor prognosis in resectable pancreatic cancer were determined. The hazard ratios of the indicators were eventually examined under the stratification of patients’ clinical stages.

**Results:**

A total of 202 patients were involved in the study. The optimum cut-off values for CA19-9 and CA19-9/TB for predicting overall survival were 219.4 (p = 0.0075) and 18.8 (p = 0.0353), respectively. CA19-9>219.4 increased the risk of patient mortality by 1.70 times (95% CI 1.217-2.377, p = 0.002), and tumor poor differentiation raised the risk by 1.66 times (95% CI 1.083-2.553, P = 0.02). Based on clinical stage stratification, we found discrepancies in the predictive efficacy of CA19-9 and CA19-9/TB. CA19-9 was a better predictor in clinical stage 1 (HR = 2.056[CI 95%1.169-3.616], P = 0.012), whereas CA19-9/TB indications were better in stages 2 (HR = 1.650[CI 95%1.023-2.662], P = 0.040) and 3 (HR = 3.989[CI95%1.145-13.896], P = 0.030).

**Conclusions:**

CA19-9, CEA, and tumor differentiation are predictors for patients with resectable PDAC. CA19-9 values can be adjusted based on clinical stage and bilirubin levels to better predict overall survival in patients with resectable PDAC. CA19-9>219.4 predicted poor survival in individuals in clinical stage 1, whereas CA19-9/TB>18.8 predicted poor survival for individuals in stages 2 and 3.

## Introduction

The morbidity and mortality of pancreatic cancer are rising quickly over the world (1). Non-surgical treatments for pancreatic cancer are unsatisfactory (2). The only method to achieve a radical cure is by surgical resection. Whether or not the operation is performed influences the patient’s overall prognosis and treatment strategy (3). Therefore, it is important to investigate the prognostic indicators of pancreatic cancer surgery.

Many studies have been undertaken in order to identify the best serum biomarkers for predicting the prognosis of pancreatic ductal adenocarcinoma (PDAC) patients. Previous research has shown that carbohydrate antigen 19-9 (CA19-9) is a biomarker for recurrence and survival (4). Bilirubin, on the other hand, has an effect on the level of CA19-9. Under normal conditions, bilirubin is generated from the hemoglobin of senescent erythrocytes (5). Bilirubin levels rise dramatically when the obstruction is caused by malignant disease. CA19-9 levels are higher in hyperbilirubinemia patients, resulting in a lower specificity of CA19-9 in predicting patients’ survival. This interferes with the prognostic value of CA19-9 in individuals with pancreatic cancer who have obstructive jaundice. Researchers use a variety of adjustment formulae to optimize the indicators to increase the prediction accuracy of CA19-9. The clinical stage of the patient can indicate the size of the tumor and may suggest the degree of biliary obstruction (4). In the light of this hypothesis, we expect that adjusting CA19-9 values with clinical staging and bilirubin provides a better accurate prognostic expectation than CA19-9 alone.

In this study, we reviewed the patient data of our hospital and investigated the correlation between indicators and the overall survival (OS) as well as disease-free survival (DFS) of patients in different clinical stages. It is favorable to the future clinical application of these indicators in order to improve the surgeon’s ability to predict PDAC patients prior to surgery. In compliance with the STROBE reporting checklist, we provide the following article (6).

## Materials and methods

### Patients

Between August 2009 and May 2017, we analyzed the data of PDAC patients who underwent radical surgical treatment at our institution (West China Hospital of Sichuan University, Sichuan, China). The hospital’s medical record system was used to collect patient information, laboratory and pathological features for this study. All patients did not get preoperative pretreatment but received postoperative adjuvant chemotherapy (based on S1 or gemcitabine). The patient’s latest follow-up was in April 2022. The study was conducted in accordance with the Declaration of Helsinki (as revised in 2013). This study was approved by the Ethics Committee of West China Hospital of Sichuan University, and written informed consent was obtained from all patients before surgery.

Eligibility criteria: (I) patients with PDAC who underwent radical surgical treatment between August, 2009 to May 2017; (II) no restriction was imposed on age and gender; (III) histopathological diagnosis of pancreatic ductal adenocarcinoma with intact pathological samples.

Exclusion criteria: (I) metastasis was found at the initial operation; (II) R2 resection; (III) data on clinical, laboratory characteristics, treatments, outcomes and follow-up are not available; (IV) died within 30 days; (V) lost to follow-up within two years after operation.

The OS was defined as from the dates of surgery to the dates of death. The DFS was calculated from the interval between the dates of surgery and the first recurrence or metastasis. If there was no recurrence or metastasis at the time of the patient’s death, the DFS and OS dates were the same.

### Risk factor analysis

We investigated the correlation between risk factors and the prognosis of patients in different clinical stages. The potential indicators such as age, gender, pain, degree of tumor differentiation, vascular invasion, nerve invasion, cutting edge status, tumor site, CA19-9, Carcinoembryonic antigen (CEA), total bilirubin (TB), CA19-9/TB, and clinical stage, etc. were used to identify the univariate risk factors affecting resectable PDAC patients’ OS and DFS, respectively. The eighth edition of the American Joint Committee on Cancer (AJCC) guidelines for pancreatic cancer is used to determine Tumor-Node-Metastasis (TNM) and clinical stage of malignancies (7).

On the basis of univariate test, Cox survival regression analysis was further carried out to find the risk factor and the hazard ratio (HR). The analysis is based on the premise that the Kaplan–Meier survival curve does not cross. We only investigated variables that were statistically significant after initial screening, on which HR values and P values were built.

### Correlation analysis and linear regression analysis

We performed Pearson correlation to examine the relationship between CA19-9 and tumor pathological parameters such as tumor maximum diameter, lymph node metastases, tumor site, differentiation, vascular invasion, and nerve invasion in order to improve the accuracy of CA19-9 in predicting OS. In addition, a linear regression analysis was applied to see if the indicators and CA19-9 have a linear correlation.

### Statistical methods

Continuous variables were stratified using the X-tile software (8) (version 3.6.1, Yale University, Connecticut, USA) to determine ideal cut-off values according to the minimum P values from log-rank, chi-square statistics, and convert into classified variables. Univariate risk factors were performed by Log-rank analyses. “Backward: Conditional” of Cox proportional hazards model was used for multivariate analysis and the hazard ratios were obtained. Pearson test and linear regression analysis is used to analysis the correlation between continuous variables. The Kaplan–Meier method was used to analyze survival duration. These statistical analyses were performed using IBM SPSS Statistics for Windows, version 22.0 (IBM Corp., Armonk, NY, USA). A P value of < 0.05 was considered statistically significant. All P values are derived from two-tailed tests.

## Results

### Patient characteristics

The study comprised 216 individuals who had radical excision of pancreatic ductal adenocarcinoma between August 11, 2009 and May 16, 2017. Ten patients with PDAC oligometastasis concurrent resection were eliminated, four patients with R2 resection verified by intraoperative and postoperative pathology were excluded, and no patients died within 30 days. As a result, 202 patients were included in the research. Seven patients who lived for 24-60 months but were unable to contact effectively in the most recent follow-up were included in the research as the censored value. **Table 1** summarizes the patients’ characteristics.

**Table 1:** Patient characteristics and baseline information.

Characteristic		Patients	[%]	Clinical Stage						Statistics	
		All(N=202)		I(N=80)		II(N=100)		III(N=22)		X^2^	P
Sex	Male	120	[59.4]	47	[58.8]	64	[64.0]	9	[40.9]	4.011	0.135
	Female	82	[40.6]	33	[41.2]	36	[36.0]	13	[59.1]		
Age, (range), year		60	(30-84)	59	(34-84)	62	(30-77)	56	[43-74]	4.873	0.087
Abdominal/back pain		121	[59.9]	51	[63.7]	57	[57.0]	13	[59.1]	0.850	0.654
Tumor site	Head	143	[70.8]	56	[70.0]	71	[71.0]	16	[72.7]	0.066	0.967
	Body & Tail	59	[29.2]	24	[30.0]	29	[29.0]	6	[27.3]		
CA19-9 level, (IQR), U/mL		187.4	(44.3-639.8)	139.0	(37.6-498.3)	256.1	(50.5-796.2)	203.2	(81.6-1000.0)	5.640	0.060
CEA level, (IQR), ng/mL		3.0	(1.8-5.2)	2.2	(1.6-4.1)	3.8	(2.1-5.8)	2.5	(1.5-5.8)	4.506	0.105
Total Bilirubin level, (IQR), μmol/L		21.8	(11.2-184.5)	21.5	(12.2-182.2)	24.3	(10.4-183.7)	17.3	(11.1-224.5)	1.300	0.522
Surgery	Pancreaticoduodenectomy	135	[66.8]	55	[68.7]	67	[67.0]	13	[59.1]	6.282	0.392
	Distal pancreatectomy	52	[25.7]	22	[27.5]	25	[25.0]	5	[22.7]		
	Total pancreatectomy	15	[7.4]	3	[3.8]	8	[8.0]	4	[18.2]		
Cutting edge status	R0	144	[71.3]	61	[76.3]	70	[70.0]	13	[59.1]	2.642	0.267
	R1	58	[28.7]	19	[23.7]	30	[30.0]	9	[40.9]		
T	Maximum diameter of tumor, (range), centimeter	3.5	(1.0-8.5)	3.0	(1.5-4)	4.3	(1.0-8.5)	4.0	[2.0-6.0]	NA	
	1	16	[7.9]	12	[15.0]	4	[4.0]	0	[0.0]		
	2	109	[54.0]	68	[85.0]	35	[35.0]	6	[27.3]		
	3	62	[30.7]			61	[61.0]	1	[4.5]		
	4	15	[7.4]					15	[68.2]		
N	Node, (range), number	0	(0-8)	0	(0-0)	1	(0-3)	1	(0-8)	NA	
	0	131	[64.9]	80	[100.0]	40	[40.0]	11	[50.0]		
	1	64	[31.7]			60	[60.0]	4	[18.2]		
	2	7	[3.5]					7	[31.8]		
Vascular Invasion		98	[48.5]	10	[12.5]	74	[74.0]	14	[63.6]	24.390	<0.001
Nerve Invasion		112	[55.4]	36	[45.0]	62	[62.0]	14	[63.6]	5.870	0.053
Differentiation	Low	151	[74.8]	57	[71.3]	81	[81.0]	13	[59.1]	5.447	0.066
	Intermediate/High	51	[25.2]	23	[28.7]	19	[19.0]	9	[40.9]		
Recurrence		37	[18.3]	14	[17.5]	19	[19.0]	4	[18.2]	0.067	0.967
Metastasis	Abdominal	45	[22.3]	16	[20.0]	23	[23.0]	6	[27.3]	0.587	0.746
	Chest	31	[15.3]	7	[8.8]	21	[21.0]	3	[13.6]	5.189	0.075
	Multiple	15	[7.4]	8	[10.0]	6	[6.0]	1	[4.5]	1.332	0.514
OS	median (range)	31.5	(1-120)	40	(4-120)	24	(1-94)	20.5	(8-76)	8.168	0.017
	1-year-survivor	166	[82.2]	71	[88.7]	75	[75.0]	20	[90.9]		
	3-year-survivor	119	[58.9]	56	[70.0]	52	[52.0]	11	[50.0]		
	5-year-survivor	58	[28.7]	31	[38.8]	22	[22.0]	5	[22.7]		
DFS	median (range)	19.0	(1-120)	26	(1-120)	17	(1-94)	11	(4-76)	7.293	0.026
	1-year-survivor	129	[63.9]	58	[72.5]	61	[61.0]	10	[45.5]		
	3-year-survivor	73	[36.1]	36	[45.0]	31	[31.0]	6	[27.3]		
	5-year-survivor	53	[26.2]	30	[37.5]	18	[18.0]	5	[22.7]		

OS, Overall Survival; DFS, Disease Free Survival; CA19-9, Carbohydrate Antigen 19-9; CEA, Carcinoembryonic Antigen; IQR, interquartile range.

### Cut-off value of continuous variables

**Figure 1:**
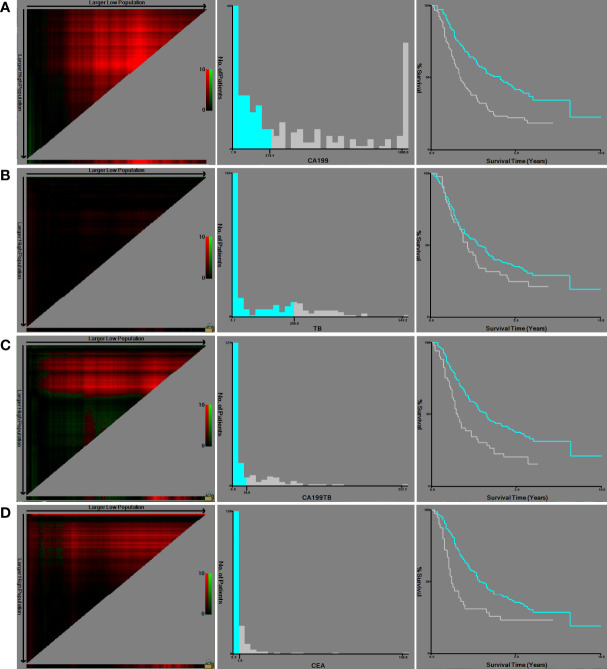
X-tile software was used to obtain the cut-off values of continuous variables and to perform survival analysis: **(A)** CA19-9; **(B)** TB; **(C)** CA19-9/TB; **(D)** CEA.

Using the X-tile program, continuous variables such as CA19-9, CEA, TB, and CA19-9/TB were stratified (**Figure 1**). OS was used as the dependent variable. Then, using the optimal cut-off value, an accurate and vital survival analysis was performed by subgroups for OS and DFS, respectively. The analysis results revealed that the appropriate cut-off values for CA19-9, CEA, TB and CA19-9/TB, were 219.4 (p = 0.008), 5.8 (p = 0.138), 200 (p = 1.000) and 18.8 (p = 0.035), respectively. The survival curves showed that patients with higher CA19-9 and CA19-9/TB values had a shorter OS and DFS.

### Risk factors analysis

**Figure 2:**
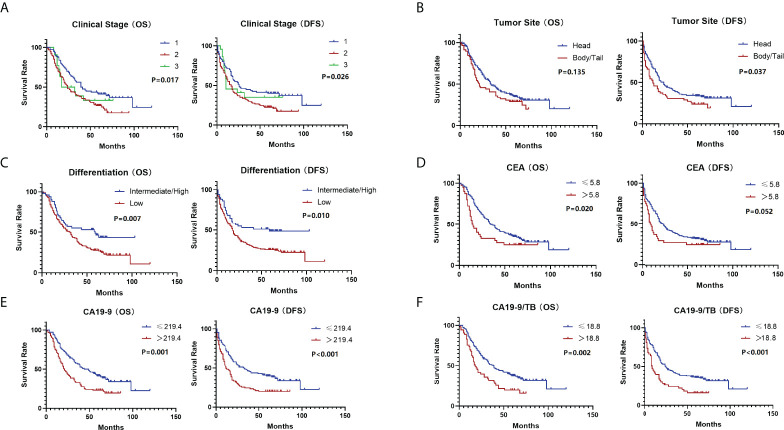
The survival curves (OS & DFS) of the indicators after primary screening: **(A)** Clinical stage; **(B)** Tumor site; **(C)** Differentiation; **(D)** CEA; **(E)** CA19-9; **(F)** CA19-9/TB.

The Kaplan–Meier analysis was carried out to screen the univariate indicators affecting OS and DFS (**Table 2**). CA19-9 was found to be statistically significant in predicting OS in a univariate survival analysis. The survival curves of the indicators with statistically significant differences were recorded in **Figure 2**. CA19-9>219.4 increased the risk of death by 1.70 times (95% CI 1.217-2.377, P = 0.002) in the Cox proportional hazards model, and Tumor low differentiation increased the risk of death by 1.66 times (95% CI 1.083-2.553, P = 0.02).

**Table 2:** Risk factors affecting survival.

Variables	OS					DFS				
	Log-Rank	P	Cox(HR)	95%CI	P	Log-Rank	P	Cox(HR)	95%CI	P
Age(>65)	1.079	0.299				1.178	0.278			
Sex(Male)	2.228	0.135				2.508	0.113			
Pain	0.272	0.602				0.074	0.786			
Differentiation(Low)	7.293	0.007	1.663	1.083-2.553	0.020	6.72	0.01	1.591	1.049-2.413	0.029
Vascular Invasion	0.042	0.838				0.038	0.846			
Nerve Invasion	2.652	0.103				3.411	0.065			
R0	0.247	0.619				0.228	0.633			
Site	2.238	0.135				4.328	0.037			
CA19-9>219.4	12.096	0.001	1.701	1.217-2.377	0.002	12.242	<0.001	1.696	1.215-2.365	0.002
CA19-9/TB>18.8	10.074	0.002				12.502	<0.001			
CEA>5.8	5.421	0.02				3.763	0.052			
TB>200	1.445	0.229				0.618	0.432			
Clinical stage	8.168	0.017				7.293	0.026			

OS, Overall Survival; DFS, Disease Free Survival; CA19-9, Carbohydrate Antigen 19-9; CEA, Carcinoembryonic Antigen; TB, Total Bilirubin.

### Correlation analysis between CA19-9 and tumor characteristics

CA19-9 has a correlation with both the maximum diameter of the tumor (P = 0.022) and the positive lymph nodes (P = 0.033) according to linear regression analysis. There is no multicollinearity between the two indicators (variance inflation factor (VIF) = 1.000). The Dubin Watson (DW) test was used to determine that the independent variables had no autocorrelation (DW value = 1.804, Adjusted R2 value=0.038). Based on the linear correlation between CA19-9 and tumor information, the correction formula CA19-9 = 160.562+46.685*d+39.654*ln (d = tumor maximum diameter, ln=lymph nodes metastases number) was developed.

### Survival analysis with adjusted CA19-9

According to the findings, tumor stage has a significant impact on patients’ overall survival. We divided patients into three groups based on their clinical stage to reduce the interference of tumor stage. The ability of CA19-9 and CA19-9/TB to predict disease progression was then tested at different stages. In clinical stage 1, the HR of CA19-9 was higher than CA19-9/TB (HR=2.056 [CI 95% 1.169-3.616], P=0.012 vs HR=1.513[CI 95% 0.802-2.856], P=0.201), while lower in stage 2 (HR=1.381[CI 95% 0.880-2.166], P=0.160 vs HR=1.650[CI 95% 1.023-2.662], P=0.040) and stage 3(HR=2.359[CI 95% 0.812-6.854], P=0.115 vs HR=3.989[CI 95% 1.145-13.896], P=0.030).

## Discussion

In this study, we first looked into the factors that affect OS and DFS, and found that CA19-9, CEA, and low-differentiation were mostly predictors for poor patient outcomes. Meanwhile, using correlation analysis, we found that CA19-9 had a possible linear association with tumor dimensions and the positive lymph nodes. CA19-9 levels may be connected to tumor growth or metastatic burden, according to this research. Despite the fact that this linear association is modest, it gives us fresh ideas. These two indications are, interestingly, closely tied to TNM staging. We hypothesized that the patient’s TNM stage could be effective in predicting the indicators’ predictive effect. We divided patients into groups based on their clinical stage based on this idea. CA19-9 and CA19-9/TB were investigated for their prognostic accuracy in range of clinical stages. CA19-9 was found to have better predictive qualities for patients’ OS when stratified by clinical stage and adjusted with TB. Briefly, we found using CA19-9>219.4 in clinical stage 1 and CA19-9/TB>18.8 in clinical stages 2 and 3 has a better effect on dividing patients’ OS as well as DFS and indicates a worse prognosis. More importantly, TNM staging is broadly assessable from pre-operative radiographic indicators. It is helpful for clinicians to make preoperative judgments on patient prognosis.

Pancreatic cancer has a dismal overall prognosis, however patient response to treatment varies substantially. Surgical operation is an important aspect of the patient’s treatment process. Patients with resectable pancreatic cancer have a significantly better prognosis than those with unresectable cancer (9). There is no doubt that patient treatment could be improved if clinicians could predict a patient’s long-term prognosis prior to surgery rather than thereafter *via* pathology. However, predicting a patient’s survival before surgery is challenging. Using CA19-9 to predict the long-term prognosis of resectable patients is a convenient and efficient method (10). It also serves as a signal for recurrence surveillance and chemotherapy sensitivity. However, CA19-9 is not specific enough because of the interference of raised bilirubin due to biliary obstruction (11). Previous investigators directly used bilirubin-corrected CA19-9 without considering clinical staging, which may be flawed (12). A bile duct obstruction, for example, would be unusual in a patient with a tiny tumor. Using CA19-9/TB directly may lead to a lower predictive value and diverge from the primary purpose in this situation.

Previously, researchers employed CA19-9 or CA19-9/TB as an indication of prognosis or tumor malignancy in earlier. Others have developed more complex compositions, such as CA19-9+Bilirubin+CA19-9/(Bilirubin^-1^) (4). To reduce the effect of biliary blockage on CA19-9, Zhao et al. developed a correction formula for CA19-9 following bile duct draining (13). Xu et al. found that tumor volume influenced CA19-9, and that volume-corrected CA19-9 may be employed as an independent risk factor influencing PDAC prognosis (14). It is apparent that utilizing more sophisticated formulas or adding more markers may increase predictive accuracy, but whether this is beneficial for clinical usage and popularization is debatable. We should find a new balance between a simple rough calculation and a complex precise one. We believe that using clinical staging to stratify patients is reasonable in this situation. Preoperative radiography can provide a rough estimate of clinical staging for patients with resectable PDAC based on tumor diameter and lymph node morphology in actual clinical practice, especially following the 8th revision of AJCC staging (15, 16). We believe that staging, in addition to the serum index CA19-9, can incorporate more preoperative information to improve patient prognosis prediction.

Other clinical and pathological variables, such as pain, cutting edge status (R0 vs. R1), vascular invasion, and nerve invasion, were found to be of limited utility in assessing prognosis in our study. Pain, as demonstrated by Xu et al., cannot be used alone to predict PDAC (5). The two most common causes of stomach and/or back pain produced by pancreatic cancer are chronic pancreatitis and tumor invasion of nerves. Due to a lack of pancreatitis symptoms, we simply looked at the relationship between pain and nerve invasion, however there was no statistical significance (P=0.566). Previous research have been divided on the impact of R0/R1 resection on patient prognosis, and there is conflicting evidence on whether R1 resection has a similar prognosis to R0 resection (17). Because this was a retrospective analysis with a long follow-up period, we concentrated on the actual outcome of patients following R1 resection who did not receive neoadjuvant chemotherapy. The findings indicated that R0 and R1 resection had little effect on patient prognosis. Datta et al. explained that this may be a surrogate for biologic aggressiveness that is unlikely to be mitigated by the extent of surgical resection (18). We believe that more studies are needed to verify the new outcomes in response to current neoadjuvant therapies. In contrast, there was an association between the lower differentiation and worse prognosis. Unfortunately, 74.8% of patients had a low differentiated tumor pathology result. In contrast, if a patient’s postoperative pathology is intermediate/high differentiated, the patient has a considerably better survival rate. Although CEA had predictive effect on OS in this study, it was not as powerful as CA19-9 and was not predictive on DFS (19). Therefore, we did not incorporate further correction for CEA. Also, we found an interesting result that patients with pancreatic body or tail tumors had a shorter DFS and were more likely to have disease recurrence but have similar OS. Erning’s study also stated that pancreatic body tail cancer is more likely to metastasize (20). However, Zheng et al. reviewed previous research and found that pancreatic head cancer, particularly in stage II, appears to have a significantly worse prognosis (21). It is uncertain if this phenomena is caused by anatomy or molecular biology. Further study is needed to elucidate the underlying mechanisms for these disparities.

The limitation of this study is that it was a retrospective study conducted at a single center. Since the cut-off value only comes from the population in this study, the cut-off value mainly shows the trend in the sample data. A bigger sample size is required for an accurate cut-off value. The time span is long, and the level of experience of surgeons varies. Patients were mostly from 5 years ago, therefore current adjuvant and neoadjuvant therapy were not used. In additional to this, we did not consider CA19-9-Low&Lewis (+) pancreatic cancer patients, which may have an uncertain impact on accuracy (22). Furthermore, we did not distinguish the tumor site because the sample size would be further lowered. Because the constraints listed above may cause bias in the results, further data from other centers is required for further validation of our findings.

Through this study, we found that CA19-9, CEA and tumor low-differentiated are markers of a poor prognosis in resectable PDAC patients. CA19-9 has a modest linear correlation with tumor maximum diameter and positive lymph nodes. Following clinical staging, we used CA19-9 to assess clinical stage 1 patients and CA19-9/TB to assess stage 2,3 patients, respectively. CA19-9>219.4 or CA19-9/TB>18.8 suggested a poor long-term prognosis for patients.

## Conclusions

CA19-9, CEA and tumor low-differentiated are predictors that affect the prognosis of resectable PDAC patients. CA19-9 values adjusted with clinical stage and bilirubin could better predict overall survival in patients with resectable PDAC. For patients in clinical phase 1, CA19-9>219.4 indicates a worse chance of survival. CA19-9/TB>18.8 predicts a worse OS in patients in stages 2 and 3. This study will help clinicians comprehend patients with high preoperative CA19-9 levels and simply probability of patient survival.

## Data availability statement

The raw data supporting the conclusions of this article will be made available by the authors, without undue reservation.

## Ethics statement

The studies involving human participants were reviewed and approved by West China Hospital of Sichuan University. The patients/participants provided their written informed consent to participate in this study.

## Author contributions

ZWW, PZ, CW, and BT conceived and designed the study. ZWW, PZ, XH, and ZHW were responsible for the collection and assembly of data, data analysis, and interpretation. ZWW, ML, and LW were involved in writing the manuscript. ZWW and BT revised the manuscript. All authors contributed to the article and approved the submitted version.

## Funding

The authors declare that the research was conducted in the absence of any funding including commercial or financial relationships that could be construed as a potential conflict of interest.

## Acknowledgments

The authors thank all medical staff who contributed to the maintenance of medical records and access to follow-up records.

## Conflict of interest

The authors declare that the research was conducted in the absence of any commercial or financial relationships that could be construed as a potential conflict of interest.

## Publisher’s note

All claims expressed in this article are solely those of the authors and do not necessarily represent those of their affiliated organizations, or those of the publisher, the editors and the reviewers. Any product that may be evaluated in this article, or claim that may be made by its manufacturer, is not guaranteed or endorsed by the publisher.
